# Effect of Induced Ventricular Fibrillation and Shock Delivery on Brain Natriuretic Peptide Measured Serially Following a Predischarge ICD Test

**Published:** 2007-10-22

**Authors:** Marco Budeus, Emanuel Salibassoglu, Anna Maria Schymura, Nico Reinsch, Heinrich Wieneke, Stefan Sack, Raimund Erbel

**Affiliations:** Department of Cardiology, West-German Heart Centre, University of Duisburg-Essen, Germany

**Keywords:** Predischarge ICD Test, Brain Natriuretic Peptide

## Abstract

**Objectives:**

Brain natriuretic peptide (BNP) was a marker for heart failure and cardiac wall tension. We analysed the trend of BNP after predischarge testing in order to get non-invasive details about the cardiac stress during predischarge testing.

**Methods:**

4-5 days after ICD implant we measured BNP, myoglobin, cardiac troponin I and creatine kinase in 20 patients before and 1, 5, 10, 20, 40, 60, 80, 100, 120 minutes and at the next day after predischarge testing. We evaluated actual values and percentage alterations of BNP.

**Results:**

BNP significantly increased with a maximum after 5 minutes (804.0 ± 803.4 vs. 475.7 ± 629.5 pg/ml, P < 0.0001) and in terms of the percentage values (100 vs. 199.4 ± 61.4 %, P < 0.0001) compared with baseline BNP. BNP decreased after that with the last significantly increased BNP value after 20 minutes (540.2 ± 604.9 vs. 475.7 ± 629.5 pg/ml, P = 0.017). We excluded a cardiac necrosis during predischarge testing because of similar values of myoglobin, cardiac troponin I and creatine kinase during the 2-hour follow-up.

**Conclusion:**

Our data showed a great increase with a doubling of BNP after 5 minutes as a result of induced ventricular fibrillation during predischarge test. This increase was not generated by myocardial necrosis but rather caused by an acute cardiac failure as a consequence of induced ventricular fibrillation in predischarge testing.

## Introduction

Clinical trials showed the efficacy of implantable cardioverter defibrillators (ICDs) for prevention of sudden cardiac death [1,2]. After the implantation of an ICD predischarge testing was performed to ensure the intraoperatively detected defibrillation threshold [3,4]. Ventricular fibrillation was induced and terminated with shock by the ICD for predischarge testing. Ventricular fibrillation caused a cardiac low output [5].

Brain natriuretic peptide (BNP) is a powerful neurohormonal predictor of the left ventricular function and prognosis and could reflect the pulmonary capillary wedge pressure [6,9]. In addition BNP had a half-life of 20 minutes and is produced in response to ventricular wall tension. Messenger ribonucleic acid is rapidly released in response to wall tension [9,10]. Prior studies showed an increase of atrial natriuretic peptide without evaluation of a further follow-up of atrial natriuretic peptide [11,12]. But the further trend of BNP was unclear which could comprise important information for post-shock pacing.

Hence, BNP is an appropriate parameter for the evaluation of cardiac stress during predischarge testing because of its physiology and timing release. Thus the purpose of our prospective study was to evaluate the trend of BNP after predischarge testing 4-5 days after ICD implant in an experimental study.

## Methods

### Study Population and ICD Systems

We included 20 consecutive patients (15 males, 5 females) with different ICDs during predischarge testing in an experimental study. All were patients admitted electively in our hospital for implantation of an ICD. Patients with a serum creatinine ≥ 1.5 mg/dl were excluded because of possible influence of BNP values [13]. The transvenous endocardial leads were inserted under fluoroscopy in all patients via the cephalic or subclavian vein and the ICD was positioned in sub pectoral position.

### Study Design

The BNP values (Triage Meter Plus®, Biosite GmbH, Willich, Germany) were measured directly before (baseline) and 1, 5, 10 and 20 minutes after predischarge testing. Furthermore BNP was measured for five half-life's (40, 60, 80, 100, 120 minutes) after predischarge testing and at the next day in the morning.

In addition myoglobin, cardiac troponin I and creatine kinase were also measured with Triage Meter Plus®. The normal values were 0 - 4.3 ng/ml for creatine kinase (detection limit 1.0 ng/ml), 0 - 107 ng/ml for myoglobin (detection limit 5 ng/ml) and 0 - 0.4 ng/ml for troponin I (detection limit 0.05 ng/ml). Blood was taken from a peripheral intravenous catheter.

The local medical ethics committee approved the study protocol and all patients gave written, informed consent before entering the study.

### Predischarge testing

Short-term anaesthesia was performed with propofol and midazolam in all patients. The patients were fasting and received no intravenous diuretic in the morning. Ventricular fibrillation was induced by T wave shock. The duration of ventricular fibrillation was defined as interval between the induction of ventricular fibrillation and the termination of ventricular fibrillation with a biphasic shock of the ICD. The programmed post shock pacing was not changed. All patients with CRT were tested in paced rhythm in biventricular mode. Patients with single or dual chamber ICD were tested with intrinsic QRS. We included only patients who were successfully defibrillated with the first initial delivered shock energy in order to consist the same condition for all patients in our study.

## Statistics

All data are presented as mean values ± standard deviation. Datasets were tested for normal distribution. The real BNP values showed a skew distribution. Here, the Mann-Whitney U test was employed. We calculated the percentage alterations of BNP for the reduction of standard deviation of the measurements because of the high standard deviation of real BNP values. The BNP value before predischarge testing was equated with 100% for evaluation of percentage alterations. For comparisons of the follow-up myoglobin, BNP (%),  cardiac troponin I and creatine kinase the two-sided Friedman ANOVA test was performed. A measure of the linear association between two variables was evaluated using Pearson's correlation coefficient; all statistical tests were two-tailed. A multivariate Cox regression analysis was performed on variables regarded as significant predictors (P < 0.1) with a univariate analysis. For all variables with at least a moderate level of association we estimated the individual odds ratios with confidence intervals. A P value < 0.05 was considered statistically significant. The statistical package that we used was SPSS 12.0 for Windows.

## Results

The demographic data are presented in Table 1. No patient was excluded from our study because in all patients ventricular fibrillation was induced by the first T wave shock and ventricular fibrillation was terminated with the first biphasic shock of the ICD. All patients were implanted an ICD for primary prevention. Fourteen patients (70%) were in NYHA III classification. Eleven patients had sinus rhythm, which was the predominant rhythm; six patients showed paroxysmal atrial fibrillation and three patients had permanent atrial fibrillation. Thirteen patients had a bundle brunch block in the ECG. Eleven of them (85%) had a left bundle brunch block and two of them (15%) had a right bundle brunch block. The mean QRS duration in the ECG was 138.4 ± 27.3 msec (intrinsic QRS in all patients). The mean serum creatinine was 1.02 ± 0.18 mg/dl.

### Predischarge testing

The results of predischarge testing are shown in Table 2. Ventricular fibrillation was induced in all patients at the first attempt of T wave shock and successfully defibrillated with the initial delivered shock energy.

### Enzyme and cardiac markers trend after predischarge testing

**Figure 1:**
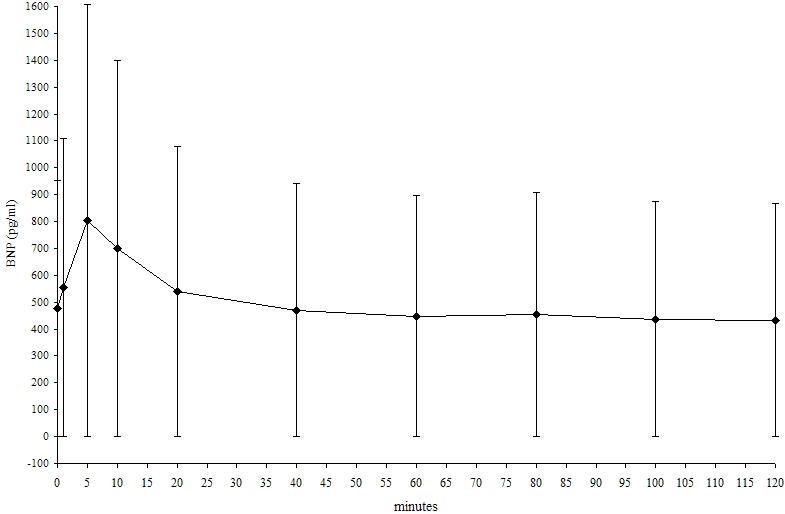
BNP trend in real values

The mean baseline BNP value was 475.7 ± 629.5 pg/ml (range 43.8 - 2860 pg/ml). The BNP five minutes after predischarge testing was significantly higher (804.0 ± 803.4 vs. 475.7 ± 629.5 pg/ml, P < 0.0001) than baseline BNP (Figure 1). The last significantly increased BNP value was measured 20 minutes (540.2 ± 604.9 vs. 475.7 ± 629.5 pg/ml, P = 0.017) after predischarge testing (Table 2).

**Figure 2:**
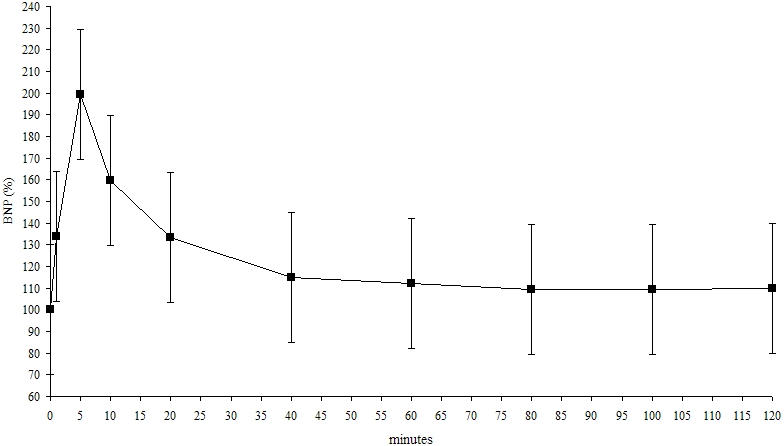
BNP trend in percentage values

BNP was doubled after 5 minutes in terms of the percentage values (100 vs. 199.4 ± 61.4 %, P < 0.0001). We also observed an increase of BNP in terms of the percentage values followed by a consistent decrease of BNP compared with baseline BNP (Figure 2).

The cardiac markers namely myoglobin, cardiac troponin I and creatine kinase showed no significant increases after predischarge testing (Table 3).

### Correlation

We did not observe any correlation (P > 0.1 for all parameters) or an independent predictor (P > 0.3 for all parameters) for the rise or the trend of BNP, myoglobin, cardiac troponin I, creatine kinase, cycle length of ventricular fibrillation, duration of ventricular fibrillation, defibrillation threshold, serum creatinine and left ventricular ejection fraction.

## Discussion

In our study we observed an increase of BNP after predischarge testing with a doubling of BNP values after five minutes. After reaching the maximum we found a consistent decrease of BNP with the last significantly increased BNP value after 20 minutes. A myocardial necrosis could be excluded by similar values of myoglobin, cardiac troponin I and creatine kinase. We did not find a correlation between the BNP trend and other parameters.

### Atrial natriuretic peptide and ventricular tachycardia

In the present study we documented a short-term increase of BNP after predischarge testing with a maximum after 5 minutes followed by a consistent decrease of BNP. As far as we know this is the first study, in which a trend of BNP for two hours after predischarge testing was examined.

Unlike prior studies [11,12], our study definitively shows a trend of BNP for a period of 120 minutes. Previous investigators reported that ventricular tachycardia caused an increase of atrial natriuretic peptide during electrophysiological study [11,12]. Fromer et al. [11] examined 11 patients and observed an increase of atrial natriuretic peptide (93 vs. 234 pg/ml, P < 0.05) 30 seconds after the induction of the ventricular tachycardia. The blood samples were taken from the right atrium [11]. Cohen et al. [12] examined 8 patients and also found an increase of atrial natriuretic peptide (95 vs. 140 pg/ml, P < 0.008) during an induced ventricular tachycardia and 30 seconds after the termination of the induced ventricular tachycardia (95 v. 145 pg/ml, P < 0.001). Cohen et al. [12] excluded a correlation between the atrial natriuretic peptide and the cycle length of ventricular tachycardia. The blood samples were taken from the right ventricle in their study [12]. Thus ventricular tachycardia caused a short-term increase of BNP and atrial natriuretic peptide as a result of atrial and ventricle wall tension [10].

### Cardiac behaviour during predischarge testing

Ventricular fibrillation causes a hypotension, a reduction of the left ventricular ejection fraction as well as a dilation of the right and left ventricle [14-16]. The cardiac stress during ventricular tachyarrhythmia was observed on the one hand with an increase of the left ventricular end-diastolic pressure [17,18] and on the other hand with an increase of BNP and atrial natriuretic peptide [11,12]. The increase of BNP or atrial natriuretic peptide was slightly temporally delayed because messenger ribonucleic acid is rapidly released in response to wall tension [11,12]. This was the possible reason for the consistent increase and maximum of the BNP level 5 minutes after predischarge testing.

Although our enzyme data excluded a myocardial necrosis during predischarge ICD test, we could not exclude a contributory role of acute ischemia during ventricular fibrillation induction in accounting for the transient rise in BNP because of high detection limit of 0.05 ng/ml for troponin I.

### Enzyme trend after predischarge testing

During the two hour follow-up we found similar values of myoglobin, cardiac troponin I and creatine kinase. An increase of myoglobin, cardiac troponin I and creatine kinase was observed in other studies [19-21]. There were important differences between these studies [19-21] and our study. Firstly, they examined patients during the implantation of an ICD. The patients received at least two shocks for the evaluation of the defibrillation threshold and in some cases an external cardioversion. Therefore a higher cumulative energy was needed for defibrillation with an energy depending increase of troponin I. In contrast to those studies our patients received only one internal shock for predischarge testing. We did not find an increase of cardiac enzymes, which was likely related to the low energy for predischarge testing in contrast to multiple shocks during ICD implantation. Secondly, ventricular fibrillation had to be induced several times during the ICD implantation as a result of multiple validations of the defibrillation threshold. Through this the time of cumulative ventricular fibrillation time increased which could cause a cardiac ischemia [19-21]. Thirdly, one or two leads were implanted during the surgery, which caused a traumatic injury of the heart. During the predischarge testing these leads were ingrown for at least four days. Thus we excluded a traumatic injury of the heart during predischarge testing in contrast to the ICD implantation. A further reason for similar values of myoglobin, cardiac troponin I and creatine kinase could be related to the sensitivity of the test and the time frame of our measurements.

## Limitation

The blood samples were taken from a peripheral intravenous catheter in our study. Therefore we could not exclude a dilution of our BNP values in contrast to the studies of Fromer et al [11] and Cohen et al. [12] Their blood samples were taken from inside the heart. BNP values showed intra-individual biological variations, which could influence our results [22]. The heterogeneous post-shock pacing might have influenced the BNP trend in our study. In other studies an increase of myoglobin, cardiac troponin I and creatine kinase was detected two hours after the defibrillation threshold validation [19-21]. Thus we could not exclude cardiac ischemia in the further follow-up or between the interval of 120 minutes and the next day. We did not find troponin I values above the detection limit of 0.05 ng/ml. Thus we could not exclude a significant alteration of troponin I below this detection limit.

## Conclusions

A doubling of BNP was detected after 5 minutes of predischarge testing as a result of induced ventricular fibrillation. This increase was generated by ventricular fibrillation and is not caused by myocardial necrosis. Through this a strong cardiac wall tension was detected indirectly with BNP as a result of induced ventricular fibrillation. But it is unclear how far a modified post-shock pacing can alter the trend of BNP. Therefore a randomized study has to be performed with different post-shock pacing intervals.

## Figures and Tables

**Table 1:**
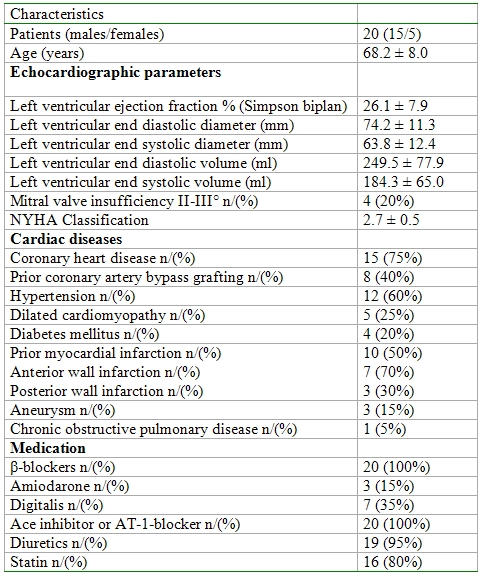
Patients characteristics

**Table 2:**
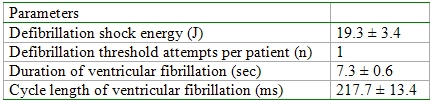
Results of predischarge testing

**Table 3:**
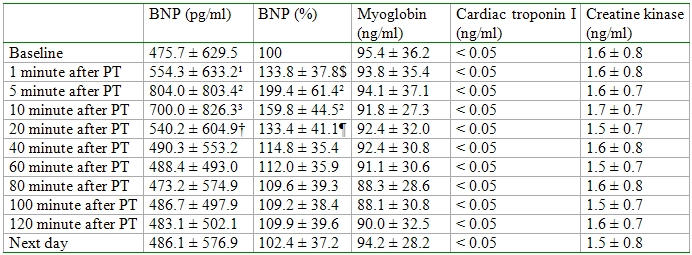
Values of BNP, myoglobin, cardiac troponin I and creatine kinase

Abbreviation: PT = predischarge testing, ¹ = P = 0.009 in comparison to BNP before predischarge testing, ² = P < 0.0001 in comparison to BNP before predischarge testing, ³ = P = 0.001 in comparison to BNP before predischarge testing, † = P = 0.017 in comparison to BNP before predischarge testing, $ = P = 0.0003 in comparison to BNP before predischarge testing, ¶ = P = 0.002 in comparison to BNP before predischarge testing

## References

[R1] Moss AJ, Multicenter Automatic Defibrillator Implantation Trial II Investigators (2002). Prophylactic implantation of the defibrillator in patients with myocardial infarction and reduced ejection fraction. N Engl J Med.

[R2] Kadish A, Defibrillators in Non-Ischemic Cardiomyopathy Treatment Evaluation (DEFINITE) Investigators (2004). Prophylactic defibrillator implantation in patients with nonischemic dilated cardiomyopathy. N Engl J Med.

[R3] Strickberger SA, Daoud EG, Davidson T (1997). Probability of successful defibrillation at multiples of the defibrillation energy requirement in patients with an implantable defibrillator. Circulation.

[R4] Marchlinski FE, Flores B, Miller JM (1988). Relation of the intraoperative defibrillation threshold to successful postoperative defibrillation with an automatic implantable cardioverter defibrillator. Am J Cardiol.

[R5] Steinbach KK, Merl O, Frohner K (1994). Hemodynamics during ventricular tachyarrhythmias. Am Heart J.

[R6] Forfia PR, Watkins SP, Rame JE (2005). Relationship between B-type natriuretic peptides and pulmonary capillary wedge pressure in the intensive care unit. J Am Coll Cardiol.

[R7] Vanderheyden M, Goethals M, Verstreken S (2004). Wall stress modulates brain natriuretic peptide production in pressure overload cardiomyopathy. J Am Coll Cardiol.

[R8] Suzuki S, Yoshimura M, Nakayama M (2004). Plasma level of B-type natriuretic peptide as a prognostic marker after acute myocardial infarction: a long-term follow-up analysis. Circulation.

[R9] Kazanegra R, Cheng V, Garcia A (2001). A rapid test for B-type natriuretic peptide correlates with falling wedge pressures in patients treated for decompensated heart failure: a pilot study. J Card Fail.

[R10] Cheung BM, Kurnana CR (1998). Natriuretic peptides-relevance in cardiac disease. JAMA.

[R11] Fromer M, Razi M, Dubuc M (1988). Effect of induced ventricular tachycardia on atrial natriuretic peptide in humans. J Am Coll Cardiol.

[R12] Cohen TJ, Liem LB (1991). Neuroendocrine response of ventricular tachycardia in humans. Am Heart J.

[R13] Tsutamoto T, Wada A, Sakai H (2006). Relationship between renal function and plasma brain natriuretic peptide in patients with heart failure. J Am Coll Cardiol.

[R14] De Piccoli B, Rigo F, Raviele A (1996). Transesophageal echocardiographic evaluation of the morphologic and hemodynamic cardiac changes during ventricular fibrillation. J Am Soc Echocardiogr.

[R15] Sylvester E, Johnson E, Hess P (2003). Defibrillation causes immediate cardiac dilation in humans. J Cardiovasc Electrophysiol.

[R16] Berg RA, Sorrell VL, Kern KB (2005). Magnetic resonance imaging during untreated ventricular fibrillation reveals prompt right ventricular overdistention without left ventricular volume loss. Circulation.

[R17] Lima JA, Weiss JL, Guzman PA (1983). Incomplete filling and incoordinate contraction as mechanisms of hypotension during ventricular tachycardia in man. Circulation.

[R18] Hachenberg T, Hammel D, Mollhoff T (1991). Cardiopulmonary effects of internal cardioverter/ defibrillator implantation. Acta Anaesthesiol Scand.

[R19] Joglar JA, Kessler DJ, Welch PJ (1999). Effects of repeated electrical defibrillations on cardiac troponin I levels. Am J Cardiol.

[R20] Schluter T, Baum H, Plewan A (2001). Effects of implantable cardioverter defibrillator implantation and shock application on biochemical markers of myocardial damage. Clin Chem.

[R21] Hurst TM, Hinrichs M, Breidenbach C (1999). Detection of myocardial injury during transvenous implantation of automatic cardioverter-defibrillators. J Am Coll Cardiol.

[R22] Wu AHB, Smith A (2004). Biological variation of the natriuretic peptides and their role in monitoring patients with heart failure. Eur J Heart Fail.

